# The Effect of Chronic Mild Stress and Escitalopram on the Expression and Methylation Levels of Genes Involved in the Oxidative and Nitrosative Stresses as Well as Tryptophan Catabolites Pathway in the Blood and Brain Structures

**DOI:** 10.3390/ijms22010010

**Published:** 2020-12-22

**Authors:** Paulina Wigner, Ewelina Synowiec, Paweł Jóźwiak, Piotr Czarny, Michał Bijak, Katarzyna Białek, Janusz Szemraj, Piotr Gruca, Mariusz Papp, Tomasz Śliwiński

**Affiliations:** 1 Laboratory of Medical Genetics, Faculty of Biology and Environmental Protection, University of Lodz, 90-136 Lodz, Poland; paulina.wigner@gmail.com (P.W.); ewelina.synowiec@biol.uni.lodz.pl (E.S.); biaalek.k@gmail.com (K.B.); 2 Department of Cytobiochemistry, Faculty of Biology and Environmental Protection, University of Lodz, 90-136 Lodz, Poland; pawel.jozwiak@biol.uni.lodz.pl; 3 Department of Medical Biochemistry, Medical University of Lodz, 90-647 Lodz, Poland; piotr.czarny@umed.lodz.pl (P.C.); janusz.szemraj@umed.lodz.pl (J.S.); 4 Biohazard Prevention Centre, Faculty of Biology and Environmental Protection, University of Lodz, 90-136 Lodz, Poland; michal.bijak@biol.uni.lodz.pl; 5 Institute of Pharmacology, Polish Academy of Sciences, 31-343 Krakow, Poland; gruca@if-pan.krakow.pl (P.G.); nfpapp@cyfronet.pl (M.P.)

**Keywords:** depression, chronic mild stress, oxidative stress, tryptophan catabolites pathway, methylation, expression, escitalopram

## Abstract

Previous studies suggest that depression may be associated with reactive oxygen species overproduction and disorders of the tryptophan catabolites pathway. Moreover, one-third of patients do not respond to conventional pharmacotherapy. Therefore, the study investigates the molecular effect of escitalopram on the expression of *Cat*, *Gpx1/4*, *Nos1/2*, *Tph1/2*, *Ido1*, *Kmo*, and *Kynu* and promoter methylation in the hippocampus, amygdala, cerebral cortex, and blood of rats exposed to CMS (chronic mild stress). The animals were exposed to CMS for two or seven weeks followed by escitalopram treatment for five weeks. The mRNA and protein expression of the genes were analysed using the TaqMan Gene Expression Assay and Western blotting, while the methylation was determined using methylation-sensitive high-resolution melting. The CMS caused an increase of *Gpx1* and *Nos1* mRNA expression in the hippocampus, which was normalised by escitalopram administration. Moreover, *Tph1* and *Tph2* mRNA expression in the cerebral cortex was increased in stressed rats after escitalopram therapy. The methylation status of the *Cat* promoter was decreased in the hippocampus and cerebral cortex of the rats after escitalopram therapy. The Gpx4 protein levels were decreased following escitalopram compared to the stressed/saline group. It appears that CMS and escitalopram influence the expression and methylation of the studied genes.

## 1. Introduction

Depression is a serious mental illness which is believed to affect 350 million people worldwide, according to the WHO. It has also been recognized as the third leading cause of disability in 2015 [1]. Although the condition affects both sexes, women are approximately twice as likely to develop symptoms [2]. If untreated, depression can lead to suicide attempts, and approximately one million people commit suicide every year [3,4]. In addition, depression is associated with high economic costs, constituting about 60% of the total cost of treating all mental conditions [5]. Unfortunately, more than a third of patients suffer from treatment-resistant depression [6].

Despite being such a serious health problem, the pathogenesis of depression remains unclear. However, previous studies suggest that disorders of the tryptophan catabolites (TRYCATs) pathway and associated overproduction of reactive oxygen species (ROS) may contribute to depression development [7,8]. Patients with depression are characterised by a decreased level of tryptophan and increased activity of IDO1 and TDO2, which converts tryptophan into kynurenine. The following stages of the TRYCATs pathway generate quinolinic acid, 3-hydroxykynurenine and 3-hydroxyanthranilic acid; these may induce the production of ROS, such as hydrogen peroxide, and increase lipid oxidation [9,10,11,12,13,14,15,16]. Indeed, patients with depression are frequently characterised by an increased level of free radicals and a decreased level of nonenzymatic antioxidants, including zinc, glutathione, and vitamins E, C, and A [17]. However, more contradictory results have been obtained with enzymatic antioxidants such as glutathione peroxidase and superoxide dismutase [18,19,20,21,22].

Depression is also associated with changes in specific parts of the brain. Interestingly, the previous study used with magnetic resonance imaging showed that depressed patients were characterised by smaller volumes of the amygdala, hippocampus, inferior anterior cingulate, and the orbital prefrontal cortex (OPFC), components of the limbic-cortico-thalamic circuit [23]. Frodl et al. (2008) confirmed that the brain of patients with depression showed more decline in grey matter density of the hippocampus, anterior cingulum, left amygdala, and right dorsomedial prefrontal cortex [24]. In addition, animal studies suggest that increased level of glucocorticoid observed in patients with depression may negatively affect neurogenesis and lead to excitotoxic damage or be associated with reduced levels of key neurotrophins in the hippocampus. Antidepressants may neutralise these effects by the increase of the neurogenesis in the hippocampus and brain-derived neurotrophic factor levels [25]. Moreover, increased levels of free radicals may cause cell death and atrophy of the neurons in the hippocampus and cortex [26]. Therefore, antidepressant drugs may exert their effectiveness by acting as antioxidants. For example, the antidepressant escitalopram, a selective serotonin reuptake inhibitor (SSRI). Escitalopram inhibits the serotonin transporter protein and is widely accepted as first-line antidepressant therapy. Escitalopram is characterized by favorable safety profile and has been shown efficacious for both acute and long-term treatments. Interestingly, escitalopram may have antioxidant properties, indicated by increased GABA levels in the frontal cortices of rats exposed to chronic mild stress (CMS) [27]. Moreover, subchronic treatment with escitalopram caused the reduced plasma SOD, CAT, malondialdehyde (MDA) and NO levels in depressed patients. However, Sarandol et al. (2007) found no difference in the levels of polyunsaturated fatty acid peroxidation products in depressed patients before and after antidepressant therapy [28]. Interestingly, the parameters came close to the results of healthy controls [29].

Therefore, the aim of the present study was to investigate the effect of chronic mild stress and antidepressant treatment with escitalopram in peripheral blood mononuclear cells (PBMCs), hippocampus, amygdala and cerebral cortex of rats. The study evaluates mRNA and protein expression, and the methylation status of gene promoters involved in oxidative stress (*Gpx1*, *Gpx4*, *Cat*, *Nos1*, *Nos2*) and the tryptophan catabolites pathway (*Tph1*, *Tph2*, *Ido1*, *Kmo*, *Kynu*). All studied gene products are presented in Table 1.

## 2. Results

### 2.1. The Effect of CMS Procedure and Escitalopram Treatment on Sucrose Intake

**Figure 1:**
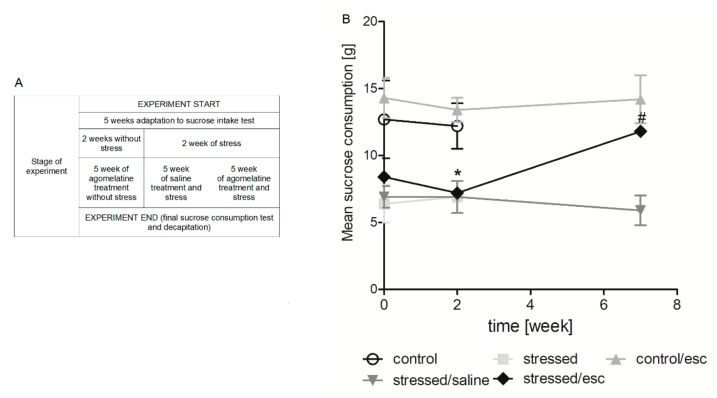
The course of the experiment of chronic mild stress and escitalopram therapy (**A**). Sucrose intake test in rats exposed to CMS for two weeks (week 2) and in animals exposed to CMS for seven weeks (week 7) and administered vehicle (1 mL/kg) or escitalopram (10 mg/kg) for five weeks (**B**). The consumption of 1.0% sucrose solution was measured in a 1-h test by weighing pre-weighed bottles. The data represents means ± SEM. n = 6. * *p* < 0.01 control group relative to the stressed group; # *p* < 0.05 stressed group relative to the stressed/esc group.

As shown in Figure 1B, after two weeks of initial stress, the stressed rats were characterised by a decrease in sucrose intake (*p* < 0.05), whereas the escitalopram-treated stressed animals showed an approximately 60% increase in sucrose intake (Week 7).

### 2.2. mRNA Expression

#### 2.2.1. Gene Expression in PBMCs

**Figure 2:**
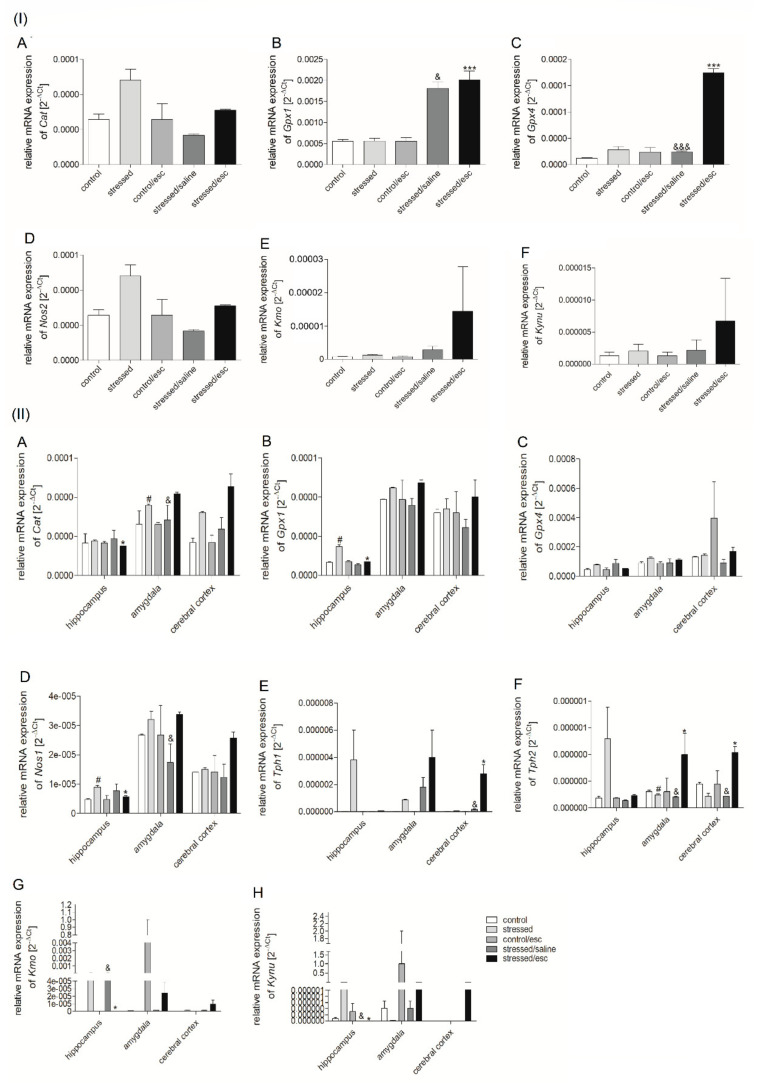
(**I**) mRNA expression of *Cat* (**A**), *Gpx1* (**B**), *Gpx4* (**C**), *Nos2* (**D**) *Kmo* (**E**), *Kynu* (**F**) in PBMCs and (**II**) mRNA expression of *CAT* (**A**), *Gpx1* (**B**), *Gpx4* (**C**), *Nos1* (**D**), *Tph1* (**E**), *Tph2* (**F**), *Kmo* (**G**), *Kynu* (**H**) in the brain structures (hippocampus, amygdala, cerebral cortex) of animals exposed to CMS for two weeks (control, stressed) and in animals exposed to CMS for seven weeks and administered vehicle (1 mL/kg) or escitalopram (10 mg/kg) for five weeks (control/esc, stressed/saline, stressed/esc). Relative gene expression levels were estimated using a 2^−ΔCt (Ctgene–Ct18S)^ method. Data represent means ± SEM. *n* = 6. * *p* < 0.05, *** *p* < 0.001 stressed group relative to stressed/esc group; # *p* < 0.05 stressed group relative to control group; & *p* < 0.05, &&& *p* < 0.001 stressed/esc group relative to stressed/saline group.

The changes in mRNA expression are presented in Figure 2. The stressed rats receiving chronic administration of escitalopram demonstrated increased *Gpx1* (H = 12.130, df = 4, *p* = 0.016, Tukey test *p* < 0.05; H = 12.130, df = 4, *p* = 0.016, Tukey test *p* < 0.001, respectively) and *Gpx4* expression in PBMCs as compared to the stressed rats and the stressed rats after chronic administration of saline (F = 129.836, df = 4, *p* < 0.001, Tukey test *p* < 0.001; F = 129.836, df = 4, *p* < 0.001, Tukey test *p* < 0.001, respectively). In the case of the *Cat*, *Nos2,* and genes involved in TRYCATs pathway, no significant differences were observed between any studied groups. 

#### 2.2.2. Gene Expression in Brain Structures

As shown in Figure 2, the effect of CMS procedure and chronic escitalopram administration on the expression of the studied genes varied according to brain structure. Reduced levels of *Cat* mRNA were observed in the hippocampus of the stressed rats after escitalopram therapy as compared to stressed animals (H = 12.233, df = 4, *p* = 0.016, Tukey test *p* < 0.05). In addition, increased *Cat* mRNA expression was observed in the amygdala after CMS (H = 12.100, df = 4, *p* = 0.017, Tukey test *p* < 0.05), as well as after chronic escitalopram administration, as compared to the stressed saline group (H = 12.100, df = 4, *p* = 0.017, Tukey test *p* < 0.05). Similarly, elevated *Gpx1* mRNA expression was recorded in the hippocampus of the stressed animals (H = 11.433, df = 4, *p* = 0.022, Tukey test *p* < 0.05), and this effect was normalised after chronic administration of escitalopram (H = 11.433, df = 4, *p* = 0.022, Tukey test *p* < 0.05). Additionally, *Nos1* mRNA expression was elevated in the hippocampus following CMS (*p* < 0.05) and this effect was normalized in stressed rats after escitalopram treatment (H = 9.462, df = 4, *p* = 0.024, Tukey test *p* < 0.05). In addition, *Nos1* was also elevated in the amygdala of stressed rats after antidepressant therapy compared to the stressed rats after saline therapy (H = 9.462, df = 4, *p* = 0.024, Tukey test *p* < 0.05).

In the case of genes involved in TRYCATs pathway, *Tph1* expression in the cerebral cortex was elevated following escitalopram treatment compared to the stressed group (H = 12.433, df = 4, *p* = 0.014, Tukey test *p* < 0.05) and the stressed group receiving saline treatment (H = 12.433, df = 4, *p* = 0.014, Tukey test *p* < 0.05). In contrast, *Tph2* mRNA expression in the amygdala fell following CMS. However, this effect was normalised by chronic administration of escitalopram (H = 8.692, df = 4, *p* = 0.034, Tukey test *p* < 0.05). In addition, higher *Tph2* mRNA expression was observed in the amygdala of rats after antidepressant therapy than in those after saline treatment (H = 8.692, df = 4, *p* = 0.034, Tukey test *p* < 0.05). Elevated *Tph2* expression was also observed in the cerebral cortex of animals after escitalopram treatment as compared to stressed group and animals after saline treatment (F = 7.123, df = 4, *p* = 0.006, Tukey test *p* < 0.05). The chronic administration of escitalopram caused a decrease of *Kmo* (H = 9.688, df = 4, *p* = 0.046, Tukey test *p* < 0.05) and *Kynu* mRNA (H = 10.937, df = 4, *p* = 0.027, Tukey test *p* < 0.05) expression in the hippocampus as compared to saline treatment.

#### 2.2.3. The Effect of Escitalopram Treatment on Gene Expression in PBMCs and Brain Structures

Escitalopram caused an increase in *Tph2* expression in the amygdala and cerebral cortex compared to PBMCs (*p* < 0.001) (Supplementary Figure S1). No significant differences were found between any of the studied groups regarding the effect of escitalopram treatment on the expression of the other studied genes involved in oxidative stress and the TRYCATs pathway.

### 2.3. Methylation Status

#### 2.3.1. Methylation Status of Promoter Regions in PBMCs

Interestingly, no significant change in the methylation status of the promoter regions of the studied genes was observed between the studied groups following CMS and escitalopram therapy (Supplementary Table S1).

#### 2.3.2. Methylation Status of Promoter Regions in Brain Structures

**Figure 3:**
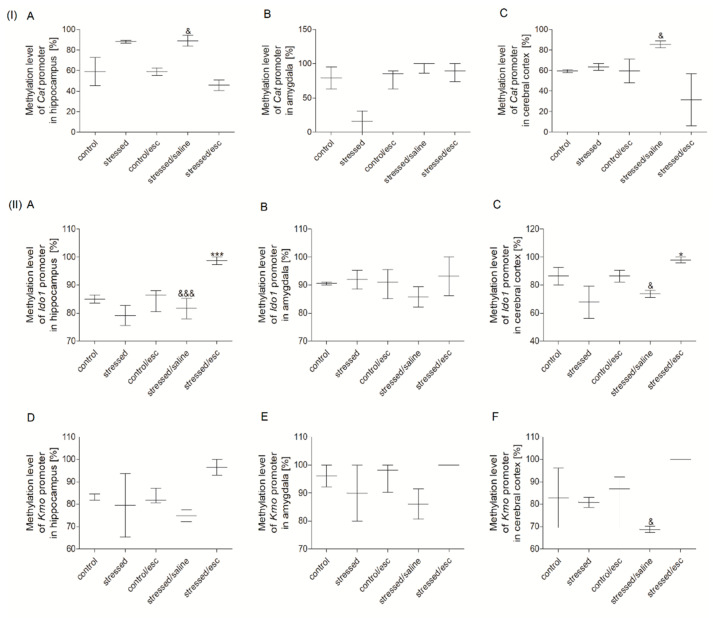
Methylation status of *Cat* promoter region (**I**) in the hippocampus (**A**), amygdala (**B**) and cerebral cortex (**C**) *Ido1* and *Kmo* promoter regions (**II**) in the hippocampus (**A**,**D**), amygdala (**B**,**E**) and cerebral cortex (**C**,**F**) of animals exposed to CMS for two weeks (control, stressed) and in animals exposed to CMS for seven weeks and administered vehicle (1 mL/kg) or escitalopram (10 mg/kg) for five weeks (control/esc, stressed/saline, stressed/esc). Data represents median and maxiumum-minimum values. n = 6. * *p* < 0.05, *** *p* < 0.001 stressed/esc group relative to stressed group; & *p* < 0.05, &&& *p* < 0.001 stressed/esc group relative to stressed/saline group.

The methylation status of the *Cat* promoter region was reduced in the hippocampus and the cerebral cortex of the stressed group after escitalopram treatment as compared to the stressed group after saline administration (H = 11.412, df = 4, *p* = 0.022, Tukey test *p* < 0.05) (Figure 3). Moreover, the methylation status of the *Ido1* promoter region in the hippocampus and the cerebral cortex was increased in the escitalopram-treated group compared to the stressed group and saline-treated group (F = 18.681, df = 4, *p* < 0.001, Tukey test *p* < 0.001; H = 12.247, df = 4, *p* = 0.016, Tukey test *p* < 0.05, respectively). In addition, the *Kmo* promoter region demonstrated increased methylated status in the cerebral cortex of stressed animals after escitalopram treatment compared to the stressed rats after saline therapy (H = 9.829, df = 4, *p* = 0.043, Tukey test *p* < 0.05). No significant changes in the methylation status of promoter regions were observed for the other studied genes (Supplementary Table S1).

#### 2.3.3. The Effect of Escitalopram Treatment on the Methylation Status of Promoter Regions in PBMCs and Brain Structures

No significant differences were found between the studied groups with regard to the effect of escitalopram treatment on methylation status of the promoter regions of all studied genes involved in oxidative stress and the TRYCATs pathway (Supplementary Figure S2).

### 2.4. Protein Expression in Brain Structures

**Figure 4:**
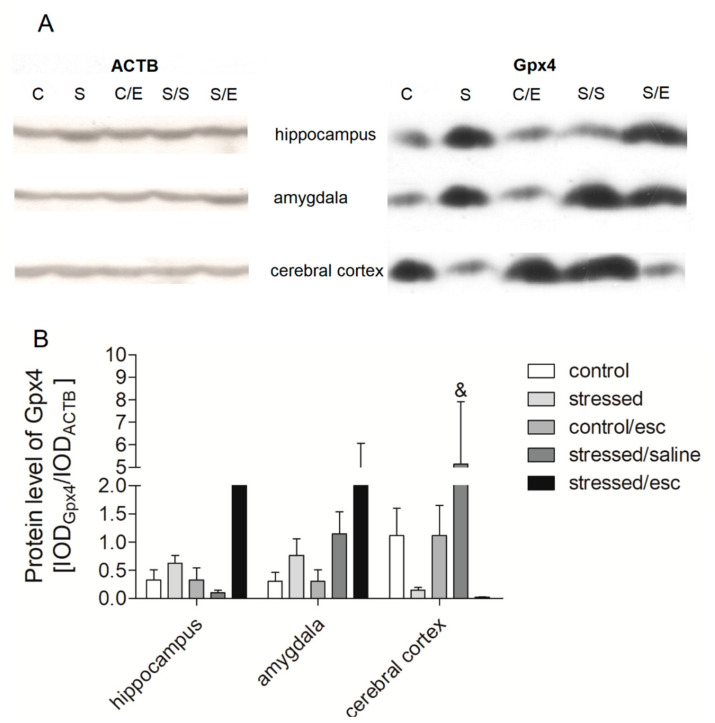
Protein expression of Gpx4 in brain structures of animals exposed to CMS for two weeks (control, stressed) and in animals exposed to CMS for seven weeks and administered vehicle (1 mL/kg) or escitalopram (10 mg/kg) for five weeks (control/esc, stressed/saline, stressed/esc). (**A**) Representative western blot analysis in midbrain and cerebral cortex. C–controls, S–stressed for two weeks, C/E–control/escitalopram, S/S–stressed/saline, S/E–stressed/escitalopram. (**B**) Levels of Gpx4 proteins measured in hippocampus, amygdala and cerebral cortex. Samples containing 25 μg of proteins were resolved by SDS-PAGE. The intensity of bands corresponding to Gpx4 was analysed by densitometry. Integrated optical density (IOD) was normalized by protein content and a reference sample (see the Methods Section for details). The graphs show the mean IODs of the bands from all analysed samples. The IOD_gene_/IOD_ACTB_ method was used to estimate the relative protein expression levels in the analysed samples. Data represent means ± SEM. n = 6. & *p* < 0.05 for the difference between stressed/saline and stressed/escitalopram groups.

In the brain structures, the only change observed in protein expression associated with escitalopram treatment was observed in the cerebral cortex, where the level of Gpx4 protein was reduced compared to stressed animals after saline treatment (H = 11.445, df = 4, *p* = 0.022, Tukey test *p* < 0.05) (Figure 4; Supplementary Figure S3). No other significant differences were observed between the studied groups regarding the other genes involved in oxidative stress and the TRYCATs pathway.

## 3. Discussion

Our findings demonstrate the impact of CMS and antidepressant treatment with escitalopram on the level of protein and mRNA expression in PBMCs and the hippocampus, amygdala and cerebral cortex. We also elucidate the methylation status of the promoter regions of the genes involved in oxidative and nitrosative stress, as well as the TRYCATs pathway. However, we didn’t evaluate any oxidative and nitrosative stress markers, including MDA, 8-oxoguanine, and 8-iso-prostaglandin F2α. The increased levels of the above-mentioned markers have been repeatedly confirmed in studies involving humans and animals. Therefore, we focused our research on an attempt to explain the molecular causes of changes in the levels of oxidative stress markers, i.e., changes in methylation and expression of genes encoding antioxidant defence enzymes [30,31,32,33].

In the presented study, the chronic mild stress was used as an animal depression model. Previous studies have shown that the CMS model is associated with the development of depression-like behaviour; one such form of behaviour is anhedonia, which is manifested in a reduction of 1% saccharose solution consumption [34,35,36,37,38]. Similarly, the present results indicate that the stressed rats demonstrated decreased consumption of sucrose solution and were hence deficient in sensitivity to rewards. On the other hand, five-week antidepressant therapy with escitalopram caused this effect to be normalised in the stressed rats. Unfortunately, there is no ideal animal model for depression and also the one used in this study has its limitations and disadvantages. The CMS model used in the presented research is based on the reflection of only one symptom of depression, anhedonia. Thus, the model is rated low in terms of the face validity whereas the criteria of the construct validity and predictive validity are highly rated. Moreover, in the case of animal models of depression, it should be remembered that some symptoms cannot be obtained in rats, e.g., suicidal thoughts. Due to the multifactorial nature of depression and the variety of psychological symptoms, each animal model used has some imperfections. In the future, the presented study results may be used to develop new animal models that better reflect depression [39].

As well as changes in sensitivity to reward, the application of CMS and escitalopram therapy resulted in changes in the expression and methylation status of promoter regions of genes involved in oxidative and nitrosative stress, as well as the TRYCATs pathway. Similarly, previous animal studies suggest that such disorders may contribute to the development of depression [40,41,42,43]. In addition, patients with depression also demonstrate exacerbation of the oxidative process and insufficient antioxidant response, as well as overproduction of neurotoxic tryptophan metabolites such as quinolinic acid and 3-hydroxykynurenine [44,45]. Interestingly, the metabolites of the TRYCATs pathway may also induce the generation of reactive oxygen species [9,10,11,12,13,14,15,16]. In addition, CMS and venlafaxine treatment, an antidepressant belonging to the serotonin–norepinephrine reuptake inhibitor group, has also been found to influence the expression and methylation of genes involved in the TRYCATs pathway, and on oxidative stress and nitrosative stress [43]. The present study continues this line of research on the impact of antidepressant treatment on changes at the molecular level.

The first key finding of the current study is that escitalopram treatment increased *Gpx1*, *Gpx4*, and *Nos2* mRNA expression in PBMCs. Enzymes encoded by *Gpx1* and *Gpx4* genes catalyse the reduction of hydrogen peroxide to water whereas *Nos1* and *Nos2* takes part in NO synthesis [46,47]. Thus, the results suggest that antidepressant treatment with escitalopram may be associated with the reduction of intensifying of the oxidative stress process or elevated expression of genes encoding antioxidant enzymes in PBMCs. In contrast, *Cat*, *Gpx1*, and *Nos1* mRNA expression was increased in the hippocampus of rats after CMS, and this effect was normalised by chronic administration of escitalopram. Moreover, the stressed animals were found to demonstrate decreased protein expression of Gpx4 in the cerebral cortex after escitalopram treatment. Similarly, stressed rats were previously found to display decreased *Gpx1* mRNA expression in the hippocampus after antidepressant therapy with venlafaxine [37]. In addition, superoxide generation was found to be increased in the cerebral cortex and hippocampus of stressed rats [48]. Such intensification of oxidative stress has been found to cause increased lipid peroxidation in the cerebral cortex and hippocampus and protein peroxidation in the cortex of stressed animals [49]. Therefore, the increased mRNA expression of *Gpx1* observed in the hippocampus of rats after CMS observed in the present study may be associated with an intensification of antioxidant defence in response to the development of oxidative stress, as indicated previously [42]. Elsewhere, *Nos1* and *Gpx1* mRNA expression in the midbrain and basal ganglia were found to be increased in stressed rats, while this effect was normalised by antidepressant therapy with venlafaxine [42]. 

Another animal study found that an oxidative imbalance causes an increase of ROS levels, resulting in disturbed *Tph1/2* mRNA expression and the reduction of serotonin synthesis [50]. Similarly, our study confirmed that CMS caused a decrease of *Tph2* mRNA expression in the amygdala. Tph converts tryptophan into 5-hydroxytryptophan and determines the concentration of serotonin [51,52]. Thus, increased expression of *Tph1* and *Tph2* can provide an adequate level of serotonin synthesis [53]. Our data confirms that antidepressant therapy with escitalopram caused an increase of *Tph1* mRNA expression in the cerebral cortex of the stressed rats, as well as *Tph2* mRNA in the amygdala. This is an important finding in the light of the “serotonin hypothesis” of clinical depression, first proposed 50 years ago. Previous studies have found an impairment of serotonin function to play a crucial role in the pathophysiology of depression.

Depression may also be associated with elevated levels of the neurotoxic metabolites of the TRYCATs pathway, such as quinolinic acid, 3-hydroxykynurenine, and 3-hydroxyanthranilic acid. The former is a toxic compound that may induce the generation of ROS oxidation [9,10,11,12,13,14,15,16]. 3-hydroxykynurenine is a product of the reaction catalysed by Kmo while 3-hydroxyanthranilic acid is formed in a reaction catalysed by Kynu [54]. Thus, depression may be associated with increased levels of Kmo and Kynu, which may act as target for antidepressant therapy [55]. Moreover, our present study confirmed that the mRNA expression of Kmo and Kynu is decreased, while the methylation status of the *Kmo* promoter region is increased, in the cerebral cortex of stressed rats after escitalopram treatment. 

Regarding *Ido1*, the gene encoding the rate-limiting enzyme of tryptophan metabolism and catalysed the oxidation of L-tryptophan to N-formylkynurenine [56], increased activity has been observed in the plasma of patients with depression and rats with anhedonia [57]. Our present findings indicate that the stressed rats demonstrated increased methylation status in the *Ido1* promoter region after escitalopram therapy, which can limit *Ido1* mRNA expression.

## 4. Conclusions

Our findings confirm that the depression and antidepressant therapy may be associated with the disorders of the interrelated biochemical pathways, including oxidative and nitrosative stress, as well as the TRYCATs pathway. Disorders in the TRYCATs pathway may induce oxidative stress processes and vice versa. In addition, it was found that the changes in mRNA and protein expression and methylation status of promoter regions appear to be dependent on the type of the tissue or brain structure. Our observations demonstrate that analyses of mRNA and protein expression, and promoter methylation status, can shed light on the mechanisms of depression development and the action of drugs for antidepressant therapy. Unfortunately, our study has some limitations. First, our research focused only on the expression and methylation of selected genes related to oxidative stress. Therefore, it cannot be conclusively assessed if escitalopram has any effects on oxidative stress and damage. Further analyses should be performed to support this hypothesis. Secondly, it has to be stressed that the depression model used is based only on anhedonia, expressed by reducing sucrose intake. Therefore, studies with other animal models of depression should also be conducted in the future.

## 5. Materials and Methods

### 5.1. Animals

Male Wistar Han rats (Charles River, Lindau/Bodensee, Germany), weighing approximately 200–220 g, were individually housed under standard conditions, i.e., room temperature (22 °C) with twelve-hour cycles of day and night, with unlimited access to food and water. Each studied group consisted of six animals. All experimental procedures were carried out in accordance with the rules of the 86/609/EEC Directive and were approved by the Local Bioethics Commission of the Institute of Pharmacology, Polish Academy of Sciences in Krakow.

### 5.2. CMS

In the first stage, rats were trained to consume a 1% solution of sucrose for lasted 1 h following 14 h water and food deprivation. After the adaptation and training period, the animals were divided into two matched groups and the sucrose intake test was performed weekly until the end of the experiment. One group of rats was exposed to a CMS procedure for two or seven weeks. The stress stimuli consisted of two periods of food or water deprivation, two periods of low-intensity stroboscopic illumination (150 flashes/min), two periods of 45° cage tilt, one period of paired housing, two periods of soiled cage (250 mL water in sawdust bedding), two periods of intermittent illumination (lights on and off every two hours), and three periods of no stress. The stressors were used individually and continuously, day and night, for 10–14 h periods. Non-stressed animals had unlimited access to food and water and were individually housed in cages without contact with the stressed animals. After two weeks of the CMS procedure, the animals were divided into two groups: one group was sacrificed and decapitated and the other was further divided into matched subgroups. The latter received daily administration of either vehicle (1 mL/kg, IP) or escitalopram (10 mg/kg, IP) for five weeks. After this five-week period and tha last sucrose intake test, all animals were sacrificed and decapitated. The scheme of the CMS procedure was presented in the Figure 1A.

### 5.3. Drug

Escitalopram (Carbosynth Ltd., Compton, Berkshire, United Kingdom), obtained commercially, was dissolved in 0.9% sterile saline and then injected at a dose of 10 mg/kg, IP (1 mL/kg of body weight).

### 5.4. Collection of PBMCs and Brain Structure Specimens

Blood samples were taken into vacutainer tubes with anticoagulants. PBMCs were isolated using Gradisol L (Aqua-Med, Lodz, Poland) and centrifugation and 400× *g* for 30 min at 4 °C. After PBMC isolation, the cell pellets were stored at −80 °C until required. In the case of the brain structures, the samples were frozen in liquid nitrogen and stored at −80 °C until required. The frozen brain samples were then suspended in PBS and manually homogenised using a FastGene^®^ Tissue Grinder (Nippon Genetics Europe, Düren, Germany). The samples of PBMC pellets and brain homogenates were used for later experiments, including RNA and DNA isolation.

### 5.5. Determination of mRNA Expression Level in PBMCs and Brain Structures

The total RNA samples were isolated from PBMCs pellets and frozen brain structures using commercial kits (GenElute Mammalian Total RNA Miniprep Kit, Sigma-Aldrich, ISOLATE II RNA/DNA/Protein Kit, Bioline, respectively) according to the manufacturer’s protocol. The quantity and purity of RNA samples were confirmed spectrophotometrically using a Synergy HTX Multi-Mode Microplate Reader, equipped with a Take3 Micro-Volume Plate (BioTek Instruments, Inc., Winooski, VT, USA).

The RNA samples were diluted to 5 ng/µl and transcribed into cDNA using a High-Capacity cDNA Reverse Transcription Kit (Applied Biosystems, Foster City, CA, USA) according to the manufacturer’s recommendations. mRNA expression was determined by real-time PCR using a TaqMan Universal Master Mix, no UNG and species-specific TaqMan Gene Expression Assay–assay ID: Rn00560930_m1 (*Cat*), Rn00577994_g1 (*Gpx1*), Rn00820818_g1 (*Gpx4*), Rn00583793_m1 (*Nos1*) and Rn00561646_m1 (*Nos2*) (Thermo Fisher Scientific, Waltham, Massachusetts, USA) according to the manufacturer’s instructions. Real-time PCR runs were performed using a CFX96TM Real-Time PCR Detection System Thermal Cycler (Bio Rad Laboratories Inc., Hercules, CA, USA).

The relative levels of mRNA expression of all studied genes were estimated as fold = 2^−ΔCt^, where ΔCt sample = Ct _target gene_ − Ct _reference gene_, with the *18S* (18S ribosomal RNA) gene being used as the reference gene. Additionally, the 2^−ΔΔCt^ method was also used to estimate the fold change in expression caused by antidepressant administration [58].

### 5.6. Determination of Methylation Status in PBMCs and Brain Structures

The DNA samples were extracted from PBMCs using QIAamp DNA Mini Kit (Qiagen, Hilden, Germany) and from brain structures using ISOLATE II RNA/DNA/Protein Kit (Bioline Ltd., London, UK) following the manufacturer’s instructions. The quantity and purity of DNA samples were estimated spectrophotometrically using a Synergy HTX Multi-Mode Microplate Reader, equipped with a Take3 Micro-Volume Plate (BioTek Instruments, Inc., Winooski, VT, USA). The DNA was then modified by bisulfite using a CiTi Converter DNA Methylation Kit (A&A Biotechnology, Gdynia, Poland) as indicated by the manufacturer. The primers for promoter regions that included CpG islands were designed using MethPrimer (http://www.urogene.org/methprimer2/) according Wojdacz et al. [59].

PCR amplification and MS-HRM assay were performed on the Bio-Rad CFX96 Real-Time PCR Detection System (BioRad Laboratories Inc., Hercules, CA, USA) equipped with Bio-Rad Precision Melt Analysis Software (BioRad Laboratories Inc., Hercules, CA, USA) to analyse the methylation status of all studied genes. The PCR reactions contained 5 × HOT FIREPol^®^ EvaGreen^®^ HRM Mix (no ROX) (Solis BioDyne Tartu, Estonia), 500 nM of forward and reverse primers, DNA samples (10 ng/µL) after bisulphite modification and PCR-grade water. The sequences of the primers and conditions of reaction are presented in Table 2. Finally, the methylation status of the studied samples was estimated based on HRM profiles obtained from the amplification of methylated template DNA (CpGenomeTM Rat Methylated Genomic DNA Standard, Merck Millipore Burlington, MA, USA) and unmethylated DNA (CpGenomeTM Rat Unmethylated Genomic DNA Standard, Merck Millipore Burlington, MA, USA). Therefore, serial dilutions of template DNA were prepared: 0%, 10%, 25%, 50%, 75%, and 100% methylated DNA.

### 5.7. Determination of Protein Expression in the Tested Brain Structures

The frozen brain tissue samples were lysed and sonicated in RIPA buffer containing serine protease inhibitor (1mM phenylmethylsulfonyl fluoride), and then centrifuged for 5 min at 5000 rpm in 4 **°**C. The concentration of protein samples was determined by the Lowry procedure using a Synergy HTX Multi-Mode Microplate Reader, equipped with a Take3 Micro-Volume Plate (BioTek Instruments, Inc., Winooski, VT, USA). Following this, 25 µg of the protein samples were separated by 10% SDS polyacrylamide gels electrophoresis and transferred onto Immobilon-P membrane (Millipore, Bedford, MA, USA). After transfer, the membranes were blocked in 5% non-fat dry milk solution and incubated with primary antibodies. Next, horseradish peroxidase-conjugated secondary antibodies were used to detect primary antibodies. A fuller description of the antibodies and incubation conditions is presented in Table 3. Finally, peroxidase substrate solution (Thermo Fisher Scientific, Waltham, MA, USA) was used for X-ray film visualization by enhanced chemiluminescence. The density of the bands was analysed using Gel-Pro^®^ Analyzer Software (Media Cybernetics Inc., Rockville, MD, USA) and normalized to β-actin levels.

### 5.8. Statistical Analysis

All statistical analyses were performed using Statistica 12 (Statsoft, Tulsa, OK, USA) and SigmaPlot 11.0 (Systat Software Inc., San Jose, CA, USA). The data were expressed as the mean ± standard error of the mean. The statistical analysis was started with the Shapiro–Wilk test which was used to evaluate data normality. Then, the one-way analysis of variance (ANOVA) was used to detect significant differences between samples with normal distribution whereas differences between probes with non-normal distribution were confirmed by the Kruskal–Wallis test. Finally, the Tukey test was used as post-hoc test. A value of *p* < 0.05 was considered to be significant.

## Figures and Tables

**Table 1:** Characteristic of all studied genes in the presented paper.

Oxidative and Nitrosative Stresses				
Gene	Enzyme	Gene Location	Function of the Enzyme	Tissue mRNA Expression
*Gpx1*	Glutathione peroxidase 1	8q32	Enzyme catalyses the reduction of organic hydroperoxides and hydrogen peroxide by glutathione, and thereby protect cells against oxidative damage.	Detected in all tissue
*Gpx4*	Glutathione peroxidase 4	7q11	Enzyme which catalyses the reduction of hydrogen peroxide, organic hydroperoxides and lipid hydroperoxides, and thereby protect cells against oxidative damage. Essential antioxidant peroxidase that directly reduces phospholipid hydroperoxide even if they are incorporated in membranes and lipoproteins (By similarity). Can also reduce fatty acid hydroperoxide, cholesterol hydroperoxide and thymine hydroperoxide.	Detected in all tissue
*Cat*	Catalase	3q32	The key antioxidant enzyme in the bodies defence against oxidative stress. Catalase is a heme enzyme that is present in the peroxisome of nearly all aerobic cells. Catalase converts the reactive oxygen species hydrogen peroxide to water and oxygen and thereby mitigates the toxic effects of hydrogen peroxide.	Detected in all tissue, however tissue enhanced–blood and liver
*Nos1*	Nitric oxide synthetase 1	12q16	Enzyme, which synthesize nitric oxide from L-arginine.	Brain, skeletal muscle
*Nos2*	Nitric oxide synthetase 2	10q25	Enzyme, which synthesize nitric oxide from L-arginine.	Detected in many tissue, however tissue enhanced–intestine, lymphoid tissue
Tryptophan catabolites pathway				
Gene	Enzyme	Gene location	Function of the enzyme	Tissue mRNA expression
*Tph1*	Tryptophan hydroxylase 1	1q22	The enzyme catalyses the first and rate limiting step in the biosynthesis of serotonin, an important hormone and neurotransmitter.	Brain, intestine, pituitary gland, stomach
*Tph2*	Tryptophan hydroxylase 2	7q22	The encoded protein catalyses the first and rate limiting step in the biosynthesis of serotonin, an important hormone and neurotransmitter.	Brain
*Ido1*	Indolamine 2,3-dioxygenasse	16q12.5	Ido1 is heme enzyme that catalyses the first and rate-limiting step in tryptophan catabolism to N-formyl-kynurenine. This enzyme acts on multiple tryptophan substrates including D-tryptophan, L-tryptophan, 5-hydroxy-tryptophan, tryptamine, and serotonin.	Blood, placenta
*Kmo*	Kynurenine 3-monooxygenase	13q25	The enzyme catalyses the hydroxylation of L-kynurenine to form 3-hydroxy-L-kynurenine. It is Required for synthesis of quinolinic acid, a neurotoxic NMDA receptor antagonist and potential endogenous inhibitor of NMDA receptor signalling.	Blood, liver, placenta
*Kynu*	Kynureninase	3q12	It is enzyme that catalyses the cleavage of L-kynurenine and L-3-hydroxykynurenine into anthranilic and 3-hydroxyanthranilic acids, respectively. Kynureninase is involved in the biosynthesis of NAD cofactors from tryptophan through the kynurenine pathway	Detected in all tissue, however tissue enhanced–blood, liver, placenta

**Table 2:** Characteristic of primer and conditions of the MS-HRM protocol.

**Oxidative and Nitrosative Stresses**											
**Gene**	**Starter Sequence (5′->3′)**	**Product Size [bp]**	**The Condition of the Reaction**								
			**The Initial Activation of the Polymerase**		**Denaturation**		**Annealing**		**Elongation**		**The HRM Analysis**
			**Temperature [°C]**	**Time [min]**	**Temperature [°C]**	**Time [s]**	**Temperature [°C]**	**Time [s]**	**Temperature [°C]**	**Time [s]**	
Cat	F: TTTGAGATTATTGTGTTTGAAA R: TACCTACACCCAAAAAAAAATA	148	95	12	95	15	59	20	72	20	Denaturation at 95 °C for 15 s, reannealing at 60 °C for 1 min and melting from 60 to 95 °C at a ramp rate of 0.2 °C
Gpx1	F: GTTGTTTTAGGTTTTGTTGTTG R: AAAACTAAAATCCTCCAACTCT	102					65				
Gpx4 (promotor 2)	F: AGGTTGGAGGTTTAGAGGTTTA R: TCCCCTAAATACAAAAATCTCT	118					59				
Gpx4 (promotor 3)	F: AGGTTGGAGGTTTAGAGGTTTA R: AAAACATAACAAAATCATCTCCC	147					65				
Nos1 (promotor 5)	F: GGGTTTTTAATTTTTTTATTGTG R: CAACCCTCATTAAAAAAACC	124					59				
Nos1 (promotor 7)	F: GTTTGAGATTGGAATTTTTTGG R: CCAAAACATCCAAAAATACACA	124					59				
**Tryptophan catabolites pathway**											
**Gene**	**Starter sequence (5′->3′)**	**Product size [bp]**	**The condition of the reaction**								
			**The initial activation of the polymerase**		**Denaturation**		**Annealing**		**Elongation**		**The HRM analysis**
			**Temperature [°C]**	**Time [min]**	**Temperature [°C]**	**Time [s]**	**Temperature [°C]**	**Time [s]**	**Temperature [°C]**	**Time [s]**	
Tph1	F: GGGAGTTTTGTTTTGGTTTTTA R: TCCTCAACCACAAAAAATCTAA	132	95	12	95	15	55	20	72	20	Denaturation at 95 °C for 15 s, reannealing at 60 °C for 1 min and melting from 60 to 95 °C at a ramp rate of 0.2 °C
Ido1	F: TTTGAGTTTTAGTGATTTTGGG R: TTAATATCTAATCCCAATCTCTAAAAC	100					59				
Tdo2 (promoter 1)	F: GATGATTTAGGTGGTTTGAGGT R: CAAAAAAAACAAAATTCATCCA	123					59				
Tdo2 (promoter 2)	F: ATGATTTAGGTGGTTTGAGGTT R: ACCCAATCTACCTAACTAACAAC	187					61.4				
Kmo	F: TTGGTTTAGGGAAGGAAATR: ATAAAAAACTAAACCCAAAACAC	150					55.7				

**Table 3:** Characteristics of antibody used in Western Blot.

**Oxidative Stress**								
**Protein**	**Primary Antibody**				**Secondary Antibody**			
	**Producent**	**The Origin of Antibodies**	**Dilution**	**Condition of Incubation [h]**	**Producent**	**The Origin of Antibodies**	**Dilution**	**Condition of Incubation [h]**
β-actin (a reference protein)	Santa Cruz Biotechnology Inc, Dallas, Texas, USA	Mouse	1:1000	1 h at room temperature	Cell Signalling Technologies Inc., Danvers, Massachusetts, USA	Anti-mouse	1:6000	1 h at room temperature
catalase	Santa Cruz Biotechnology Inc, Dallas, Texas, USA	Mouse	1:1000	overnight at 4 °C	Cell Signalling Technologies Inc., Danvers, Massachusetts, USA	Anti-mouse	1:6000	1 h at room temperature
glutathione peroxidase 4	Abcam, Cambridge, United Kingdom,	Rabbit	1:6000	overnight at 4 °C	Cell Signalling Technologies Inc., Danvers, Massachusetts, USA	Anti-rabbit	1:6000	1 h at room temperature
superoxide dismutase 1	Santa Cruz Biotechnology Inc, Dallas, Texas, USA	Mouse	1:6000	2 h at room temperature	Cell Signalling Technologies Inc., Danvers, Massachusetts, USA	Anti-mouse	1:6000	1 h at room temperature
**Tryptophan catabolites pathway**								
**Protein**	**Primary antibody**				**Secondary antibody**			
	**Producent**	**The origin of antibodies**	**Dilution**	**Condition of incubation [h]**	**Producent**	**The origin of antibodies**	**Dilution**	**Condition of incubation [h]**
Tryptophan hydroxylase 1	Cell Signalling Technologies Inc., Danvers, Massachusetts, USA	Rabbit	1:1000	overnight at 4 °C	Cell Signalling Technologies Inc., Danvers, Massachusetts, USA	Anti-rabbit	1:6000	1 h at room temperature
Tryptophan hydroxylase 2	Cell Signalling Technologies Inc., Danvers, Massachusetts, USA	Rabbit	1:6000	overnight at 4 °C	Cell Signalling Technologies Inc., Danvers, Massachusetts, USA	Anti-rabbit	1:6000	1 h at room temperature
Indoleamine 2,3-dioxygenase	Santa Cruz Biotechnology Inc, Dallas, Texas, USA	Mouse	1:1000	overnight at 4 °C	Cell Signalling Technologies Inc., Danvers, Massachusetts, USA	Anti-rabbit	1:6000	1 h at room temperature
Kynurenine aminotransferases	Santa Cruz Biotechnology Inc, Dallas, Texas, USA	Mouse	1:1000	overnight at 4 °C	Cell Signalling Technologies Inc., Danvers, Massachusetts, USA	Anti-rabbit	1:6000	1 h at room temperature
Kynureninase	Santa Cruz Biotechnology Inc, Dallas, Texas, USA	Mouse	1:1000	overnight at 4 °C	Cell Signalling Technologies Inc., Danvers, Massachusetts, USA	Anti-rabbit	1:6000	1 h at room temperature

## Supplementary Materials

Included the addition results about methylation and expression level: The following are available online at https://www.mdpi.com/1422-0067/22/1/10/s1, Figure S1: mRNA expression of Cat (A), Gpx1 (B), Gpx4 (C), Tph2 (D), Kmo (E) and Kynu (F) in PBMCs and in the brain structures of animals exposed to CMS for two weeks (control, stressed) and in animals exposed to CMS for seven weeks and administered vehicle (1 mL/kg) or escitalopram (10 mg/kg) for five weeks (control/esc, stressed/saline, stressed/esc). The effects are presented as fold change (2^−ΔΔCt^ method; Schmittgen and Livak, 2008). Data represent means ± SEM. n = 6. *** *p* < 0.001 for differences between blood and all studied brain structures. **F**igure S2. The methylation level of Tph1 (A), Ido1 (B), Kmo (C), Cat (D), Gpx1 (E), Gpx4 (F) promoter regions, Nos1 promoter 3 region (G), Nos1 promoter 7 region (H) between brain structures and PBMCs of animals exposed to CMS for two weeks control, stressed) and in animals exposed to CMS for seven weeks and treated vehicle (1 mL/kg) or escitalopram (10 mg/kg) for five weeks (control/esc, stressed/saline, stressed/esc). Data represent as means ± SEM. n = 6. **F**igure S3. Protein expression of Cat (A), Nos1 (B), Tph1 (C), Tph2 (D), Ido1 (E) and Kynu (F) in brain structures of animals exposed to CMS for two weeks (control, stressed) and in animals exposed to CMS for seven weeks and administered vehicle (1 mL/kg) or escitalopram (10 mg/kg) for five weeks (control/esc, stressed/saline, stressed/esc). Levels of Cat (A), Nos1 (B), Tph1 (C), Tph2 (D), Ido1 (E) and Kynu (F) proteins measured in hippocampus, amygdala and cerebral cortex. Samples containing 25 μg of proteins were resolved by SDS-PAGE. The intensity of bands corresponding to Gpx4 was analysed by densitometry. Integrated optical density (IOD) was normalized by protein content and a reference sample (see the Methods section for details). The graphs show the mean IODs of the bands from all analysed samples. The IODgene/IODACTB method was used to estimate the relative protein expression levels in the analysed samples. Data represent means ± SEM. *n* = 6. No significant changes were found between any groups. **T**able S1. Methylation level of, Gpx1 promoter (A), Gpx4 promoter 3 (B), Nos1 promoter 3 (C), Nos1 promoter 7 (D), Tph1 promoter (E), Ido1 promoter (F) Kmo promoter (G) in the hippocampus, amygdala, cerebral cortex and PBMCs of animals exposed to CMS for two weeks (control, stressed) and in animals exposed to CMS for seven weeks and administered vehicle (1 mL/kg) or escitalopram (10 mg/kg) for five weeks (control/esc, stressed/saline, stressed/esc). Data represents means ± SEM. n = 6. No significant changes were found between any groups.
