# Supplementary material for: The Effect of Chronic Mild Stress and Escitalopram on the Expression and Methylation Levels of Genes Involved in the Oxidative and Nitrosative Stresses as Well as Tryptophan Catabolites Pathway in the Blood and Brain Structures
Source: Int J Mol Sci. 2020 Dec 22;22(1):10. doi: 10.3390/ijms22010010 (PMC7792593; doi:10.3390/ijms22010010)
Supplement: Supplementary file 1 [file ijms-22-00010-s001.pdf]

Supplementary Material

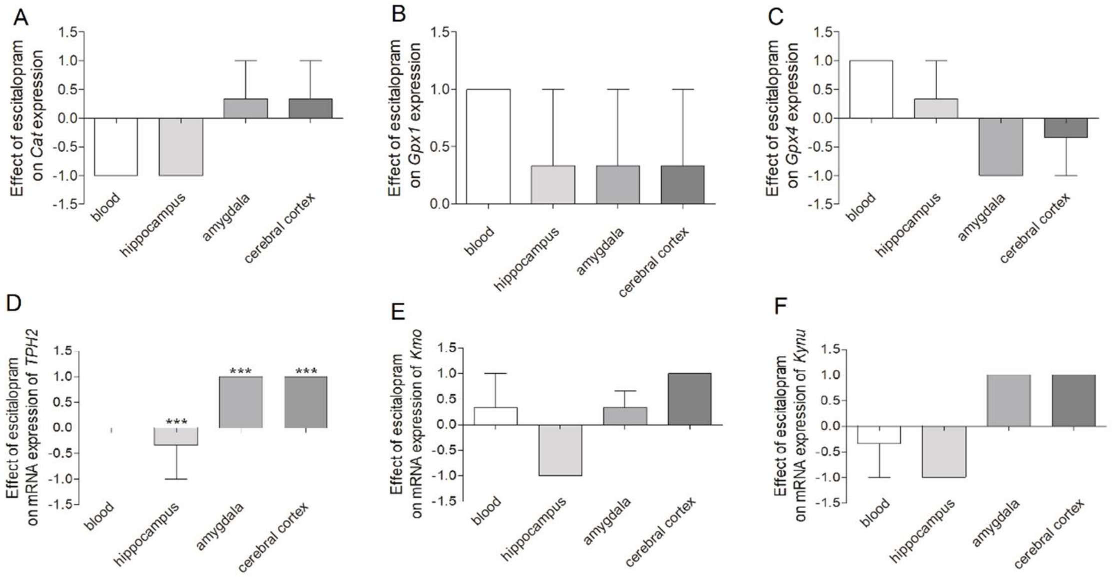

Supplementary Figure 1. mRNA expression of *Cat* (A), *Gpx1* (B), *Gpx4* (C), *Tph2* (D), *Kmo* (E) and *Kynu* (F) in PBMCs and in the brain structures of animals exposed to CMS for two weeks (control, stressed) and in animals exposed to CMS for seven weeks and administered vehicle (1 ml/kg) or escitalopram (10 mg/kg) for five weeks (control/esc, stressed/saline, stressed/esc). The effects are presented as fold change ($2^{-\Delta\Delta Ct}$ method; Schmittgen and Livak, 2008). Data represent means ± SEM. N = 6. *** $p < 0.001$ for differences between blood and all studied brain structures.

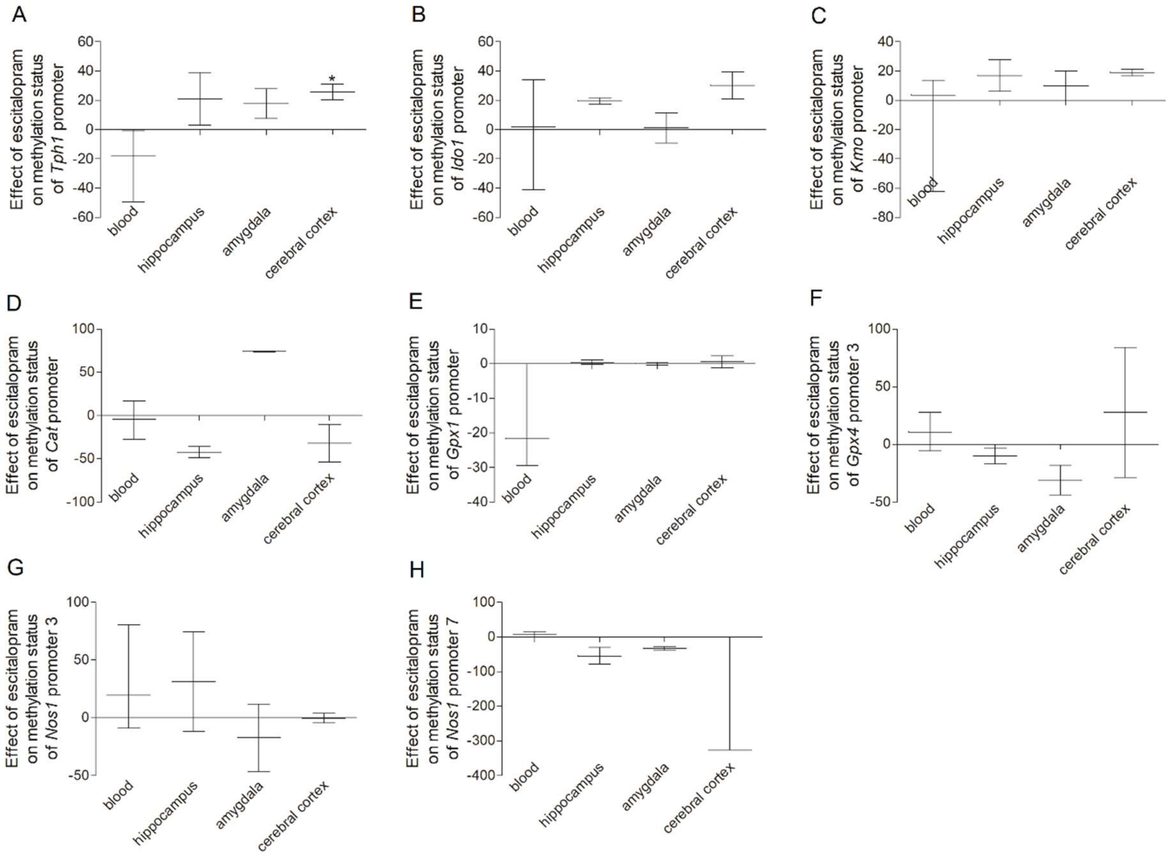

Supplementary Figure 2. The methylation level of *Tph1* (A), *Ido1* (B), *Kmo* (C), *Cat* (D), *Gpx1* (E), *Gpx4* (F) promoter regions, *Nos1* promoter 3 region (G), *Nos1* promoter 7 region (H) between brain structures and PBMCs of animals exposed to CMS for two weeks (control, stressed) and in animals exposed to CMS for seven weeks and treated vehicle (1 ml/kg) or escitalopram (10 mg/kg) for five weeks (control/esc, stressed/saline, stressed/esc). Data represent as means ± SEM. N = 6.

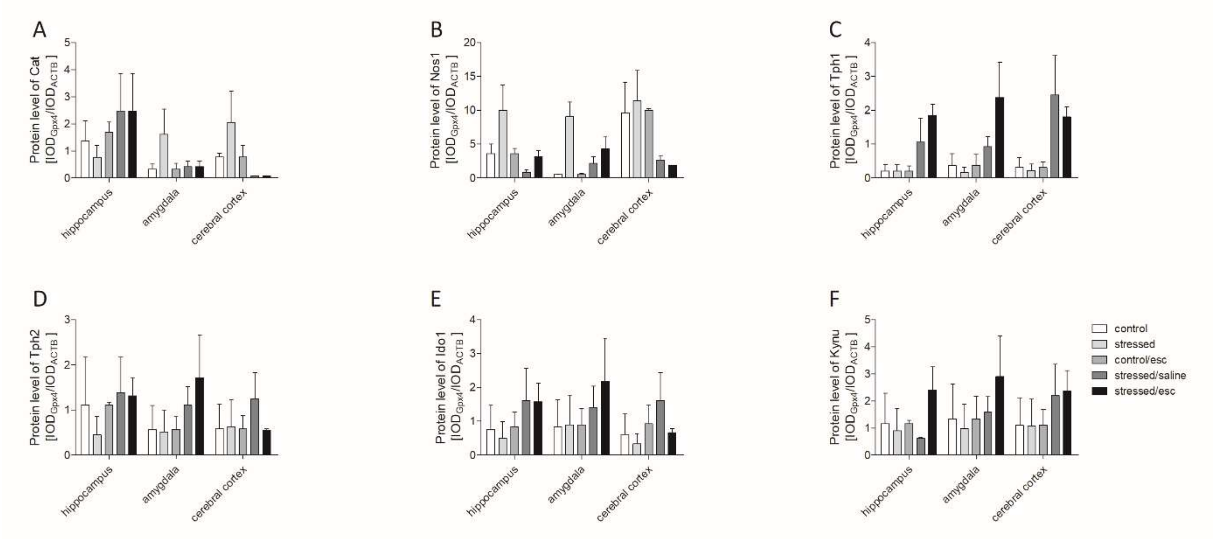

Supplementary Figure 3. Protein expression of Cat (A), Nos1 (B), Tph1 (C), Tph2 (D), Ido1 (E) and Kynu (F) in brain structures of animals exposed to CMS for two weeks (control, stressed) and in animals exposed to CMS for seven weeks and administered vehicle (1 ml/kg) or escitalopram (10 mg/kg) for five weeks (control/esc, stressed/saline, stressed/esc). Levels of Cat (A), Nos1 (B), Tph1 (C), Tph2 (D), Ido1 (E) and Kynu (F) proteins measured in hippocampus, amygdala and cerebral cortex. Samples containing 25 µg of proteins were resolved by SDS-PAGE. The intensity of bands corresponding to Gpx4 was analysed by densitometry. Integrated optical density (IOD) was normalized by protein content and a reference sample (see the Methods section for details). The graphs show the mean IODs of the bands from all analysed samples. The IOD_{gene}/IOD_{ACTB} method was used to estimate the relative protein expression levels in the analysed samples. Data represent means ± SEM. N = 6. No significant changes were found between any groups.

Supplementary Table 1. Methylation level of, *Gpx1* promoter (A), *Gpx4* promoter 3 (B), *Nos1* promoter 3 (C), *Nos1* promoter 7 (D), *Tph1* promoter (E), *Ido1* promoter (F) *Kmo* promoter (G) in the hippocampus, amygdala, cerebral cortex and PBMCs of animals exposed to CMS for two weeks (control, stressed) and in animals exposed to CMS for seven weeks and administered vehicle (1 ml/kg) or escitalopram (10 mg/kg) for five weeks (control/esc, stressed/saline, stressed/esc). Data represents means ± SEM. N = 6. No significant changes were found between any groups.

(A)

Methylation level of *Gpx1* promoter

Brain structure / PBMCs	Control	Stressed	Control/Esc	Stressed/Saline	Stressed/Esc
Hippocampus	78.90 ± 0.40	79.20 ± 0.12	78.90 ± 1.01	80.40 ± 0.12	79.60 ± 0.46
Amygdala	79.10 ± 0.06	79.70 ± 0.06	79.10 ± 2.34	79.30 ± 0.06	79.70 ± 0.17

Cerebral cortex	80.30 ± 0.52	79.90 ± 0.17	80.30 ± 1.48	76.90 ± 1.79	80.40 ± 0.81
PBMCs	90.62 ± 9.38	74.08 ± 26.21	90.62 ± 5.25	93.12 ± 3.97	57.04 ± 29.71

(B)

Methylation level of *Gpx4* promoter 3

Brain structure / PBMCs	Control	Stressed	Control/Esc	Stressed/Saline	Stressed/Esc
Hippocampus	51.30 ± 26.24	84.34 ± 1.29	51.30 ± 20.47	77.33 ± 10.57	74.18 ± 5.21
Amygdala	100.00 ± 0.00	100.00 ± 0.00	99.00 ± 0.58	100 ± 3.21	69.02 ± 7.43
Cerebral cortex	89.78 ± 0.68	48.63 ± 27.52	89.78 ± 5.09	84.15 ± 4.12	76.34 ± 5.01
PBMCs	53.32 ± 5.70	62.37 ± 7.17	53.32 ± 11.42	64.28 ± 1.98	73.31 ± 3.06

(C)

Methylation level of *Nos1* promoter 3

Brain structure / PBMCs	Control	Stressed	Control/Esc	Stressed/Saline	Stressed/Esc
Hippocampus	65.10 ± 14.70	46.29 ± 7.03	65.10 ± 8.92	67.28 ± 14.13	77.47 ± 17.96
Amygdala	50.02 ± 0.47	69.27 ± 5.38	50.02 ± 6.02	49.91 ± 0.39	52.09 ± 11.50
Cerebral cortex	64.71 ± 15.62	67.09 ± 13.59	64.71 ± 14.03	68.44 ± 9.69	66.70 ± 16.02

PBMCs	81.29 ± 2.15	46.62 ± 24.450	81.29 ± 7.90	57.97 ± 21.17	77.05 ± 16.60

(D)

Methylation level of *Nos1* promoter 7

Brain structure / PBMCs	Control	Stressed	Control/Esc	Stressed/Saline	Stressed/Esc
Hippocampus	81.53 ± 12.37	81.53 ± 12.37	80.55 ± 5.76	38.83 ± 6.66	27.30 ± 1.92
Amygdala	76.27 ± 0.23	76.27 ± 0.34	76.27 ± 8.16	12.26 ± 1.28	38.35 ± 6.39
Cerebral cortex	84.65 ± 42.32	98.15 ± 15.23	66.67 ± 33.33	26.29 ± 0.03	0.00 ± 0.00
PBMCs	53.71 ± 23.57	65.21 ± 21.91	53.71 ± 19.57	76.40 ± 23.60	93.60 ± 6.40

(E)

Methylation level of *Tph1* promoter

Brain structure / PBMCs	Control	Stressed	Control/Esc	Stressed/Saline	Stressed/Esc
Hippocampus	83.82 ± 5.30	78.91 ± 10.41	83.82 ± 9.59	69.93 ± 0.26	100.00 ± 0.00
Amygdala	87.01 ± 7.33	82.34 ± 5.90	87.01 ± 1.56	79.23 ± 2.11	98.67 ± 0.88
Cerebral cortex	74.03 ± 0.02	74.21 ± 3.18	74.03 ± 2.29	64.58 ± 6.53	99.33 ± 0.33
PBMCs	88.84 ± 6.96	85.99 ± 4.35	85.99 ± 1.45	84.11 ± 4.79	65.97 ± 10.031

(F)

Methylation level of *Ido1* promoter

Brain structure / PBMCs	Control	Stressed	Control/Esc	Stressed/Saline	Stressed/Esc
PBMCs	86.91 ± 12.85	90.65 ± 6.70	90.65 ± 1.41	88.29 ± 10.75	80.77 ± 21.43

(G)

Methylation level of *Kmo* promoter

Brain structure / PBMCs	Control	Stressed	Control/Esc	Stressed/Saline	Stressed/Esc
PBMCs	97.13 ± 1.10	84.94 ± 7.08	99.13 ± 0.92	91.94 ± 3.13	98.33 ± 0.33
